# A Tyrosine-Hydroxylase Characterization of Dopaminergic Neurons in the Honey Bee Brain

**DOI:** 10.3389/fnsys.2017.00047

**Published:** 2017-07-10

**Authors:** Stevanus R. Tedjakumala, Jacques Rouquette, Marie-Laure Boizeau, Karen A. Mesce, Lucie Hotier, Isabelle Massou, Martin Giurfa

**Affiliations:** ^1^Research Centre on Animal Cognition, Center for Integrative Biology, Centre National de la Recherche Scientifique (CNRS), University of ToulouseToulouse, France; ^2^Advanced Technology Institute in Life Sciences (ITAV), Centre National de la Recherche Scientifique—Université Paul Sabatier Toulouse III (CNRS-UPS), Université Paul Sabatier Toulouse III (UPS), Université de ToulouseToulouse, France; ^3^Department of Entomology, University of MinnesotaSaint Paul, MN, United States

**Keywords:** *Apis mellifera*, dopamine, dopaminergic signaling, neural circuits, neural clusters

## Abstract

Dopamine (DA) plays a fundamental role in insect behavior as it acts both as a general modulator of behavior and as a value system in associative learning where it mediates the reinforcing properties of unconditioned stimuli (US). Here we aimed at characterizing the dopaminergic neurons in the central nervous system of the honey bee, an insect that serves as an established model for the study of learning and memory. We used tyrosine hydroxylase (TH) immunoreactivity (ir) to ensure that the neurons detected synthesize DA endogenously. We found three main dopaminergic clusters, C1–C3, which had been previously described; the C1 cluster is located in a small region adjacent to the esophagus (ES) and the antennal lobe (AL); the C2 cluster is situated above the C1 cluster, between the AL and the vertical lobe (VL) of the mushroom body (MB); the C3 cluster is located below the calyces (CA) of the MB. In addition, we found a novel dopaminergic cluster, C4, located above the dorsomedial border of the lobula, which innervates the visual neuropils of the bee brain. Additional smaller processes and clusters were found and are described. The profuse dopaminergic innervation of the entire bee brain and the specific connectivity of DA neurons, with visual, olfactory and gustatory circuits, provide a foundation for a deeper understanding of how these sensory modules are modulated by DA, and the DA-dependent value-based associations that occur during associative learning.

## Introduction

Honey bees serve as a well-established model to understand learning and memory (Menzel, 1999, 2001; Giurfa, 2007; Giurfa and Sandoz, 2012), and a number of protocols have been developed to study the behavioral, neural and molecular correlates of such processes (Giurfa, 2007). The olfactory conditioning of the sting extension response (SER) is an important protocol that allows the study of aversive learning and memory under controlled experimental conditions (Vergoz et al., 2007; Carcaud et al., 2009; Giurfa et al., 2009; Roussel et al., 2009; Tedjakumala and Giurfa, 2013). The SER is a defensive behavior elicited in bees by potentially noxious stimuli (Breed et al., 2004). In the laboratory, it can be triggered by an electric shock delivered to a harnessed bee (Burrell and Smith, 1994; Núñez et al., 1997). Bees learn to associate this aversive electric stimulus (the unconditioned stimulus, US) with an odorant (the conditioned stimulus, CS). Furthermore, Dopamine (DA) signaling has been found to be indispensable for SER conditioning, as pharmacological blocking with different DA antagonists suppresses the capacity of bees to learn an odor-shock association through an inhibition of the aversive (US) pathway (Vergoz et al., 2007).

Recent pharmacological experiments have revealed that the role of DA in bees is more complex than just mediating aversive reinforcement (Tedjakumala et al., 2014). These experiments showed that the dopaminergic system can down-regulate the unconditioned responsiveness to electric shocks. This responsiveness is quantified by subjecting harnessed bees to a series of increasing voltages that enhance their tendency to respond with a SER. Pharmacological blockade of the dopaminergic system results in an *increase* of the responsiveness to the aversive US. It has been thus suggested that the dopaminergic system of the bee brain is functionally heterogeneous and includes at least two classes of DA neurons: one controlling global aversive responsiveness through an inhibitory action, and the other mediating aversive US signaling during aversive learning (Tedjakumala et al., 2014).

In the light of this heterogeneity, an accurate neuroanatomical characterization of DA neurons in the bee brain is warranted. This characterization should enable the identification of structures and neural modules of the bee brain that are targeted by DA neurons, thus providing the anatomical bases for associations involved in stimulus-reinforcement and for the modulation of behavioral responsiveness. Previous work performed almost three decades ago has reported the presence of putative dopaminergic neurons in the bee brain by means of immunocytochemical studies using anti-DA antisera (Schürmann et al., 1989; Schäfer and Rehder, 1989). Building on this work, we characterized the dopaminergic neurons in the central nervous system of the honey bee by immunolabeling tyrosine hydroxylase (TH), DA’s rate-limiting synthetic enzyme (Fon and Edwards, 2001). TH converts tyrosine into dihydroxylphenylalanine (L-DOPA), which is subsequently converted into DA. Thus by targeting TH we aimed at immunolabeling and analyzing neurons that synthesize DA endogenously. Our neuroanatomical data were gathered through a combination of immunocytochemistry using fluorescence-conjugated antibodies and 3D-confocal imaging of optical sections captured from whole-mounted bee brains. In this way, it was possible to reconstruct complete dopaminergic networks in the bee brain without the potential for loss of tissue regions. A complete characterization of DA neurons in the protocerebrum of *Drosophila*, at a single cell resolution, has been achieved using TH GAL4-transgene and TH antibody (Mao and Davis, 2009). To facilitate our reconstruction and identification of newly described DA processes in the bee brain, we used this characterization of dopaminergic circuits in the fruit fly brain as a reference. The comprehensive mapping of DA-synthesizing neurons in the honey bee brain sets a strong foundation for understanding the varied roles of DA in learning, memory and other associated behaviors.

## Materials and Methods

### Insects

Honey bees (*Apis mellifera*) were obtained from colonies located in the apiary of the University Paul Sabatier. Only foragers were used for this study as they have significantly higher DA levels than nurses or guards (Taylor et al., 1992). To this end, a feeder filled with 30% (weight/weight) sucrose solution was set at the apiary and true foragers were collected upon feeding.

Bees were brought to the laboratory and chilled on ice for approximately 5 min. Afterwards, they were individually harnessed in metal holders from which only the head capsule protruded. The bees were left for at least 1 h in resting conditions before dissection in order to reduce potential alterations of DA levels due to the prior handling (Chen et al., 2008).

### Dissection and Fixation

A window was cut in the upper part of the head capsule, between the compound eyes and the ocelli. The mandibles and the antennae were also removed, thus exposing the whole brain. The compactness of the hypopharyngeal glands was monitored to ensure that the bees were old enough to be considered foragers (Maleszka et al., 2009). The glands were removed to allow the fixative to access the brain optimally. The whole process lasted usually no longer than 30 s. Immediately after this, the bee was decapitated and the whole head capsule was fixed in 1% zinc-formaldehyde (ZnFA) in bee ringer (Ott, 2008) for approximately 20 h (overnight) at room temperature.

The following day, the head capsule was immersed in HEPES-buffered saline (HBS) and the brain was removed. The tracheae covering the brain were also carefully removed. The brain was rinsed three times in HBS, each time during 20 min, to remove the rests of ZnFA. Subsequently, the samples for whole-mounts were de- and rehydrated. The dehydration was done using Dent’s fixative (one part of DMSO: four parts of methanol) for 1 h, which was followed by another step in methanol for another hour and finally by rehydration in Tris buffer also for 1 h, all at room temperature.

The samples for microsections were immediately embedded in 5% low melting agarose (in phosphate buffered saline—PBS) after rinsing them three times during 20 min in HBS. Sectioning was done at 80–160 μm using a vibratome (Leica VT1000S). The sections were immediately kept in PBS for further processing.

### Immunocytochemistry

Brain slices were permeabilized and blocked in PBS solution containing 0.3% Triton X-100 and 5% normal goat serum (ngs) for 1 h. We used three primary antibodies: (i) a monoclonal antibody α-SYNORF1 raised in mouse against the *Drosophila* synapsin protein (UniProt ID: Q24546; courtesy of Prof. Erich Buchner, Würzburg); (ii) a polyclonal rabbit α-TH antibody (Merck Millipore, AB 152; UniProt ID: P04177); and (iii) a mouse monoclonal α-TH antibody (ImmunoStar, Cat# 22941). The α-SYNORF1 antibody has been used successfully in fruit flies *Drosophila melanogaster* and other invertebrates for synapsin detection (e.g., Klagges et al., 1996; Michels et al., 2005). The rabbit α-TH antibody reacts with most mammalian and many non-mammalian species, including insects. It has been successfully used to stain dopaminergic neurons in *Drosophila melanogaster* and *Caenorhabditis elegans* (e.g., Bou Dib et al., 2014; Lin et al., 2014). The mouse antibody recognizes TH across a wide variety of animal species. It has been shown to label neurons that specifically contain DA and no other amine in both insects and annelids (e.g., Mesce et al., 2001; Crisp et al., 2002).

The rabbit α-TH antibody was used for the main labeling and the mouse α-SYNORF1 for the background. After blocking, we incubated the samples with both antibodies (rabbit α-TH 1:50 and α-SYNORF1 1:50) for 48 h. We then rinsed them multiple times (10—20—30—2 × 60 min) in 0.3% Triton X-100. The secondary antibodies were Alexa Fluor® 488 α-rabbit (Invitrogen) and DyLight 649 α-mouse (Jackson ImmunoResearch) raised in goat. They were applied 1:100 for 24 h. Afterwards, the samples were again rinsed multiple times (10—20—30—2 × 60 min) in 0.3% Triton X-100. Whole-mounted samples were dehydrated in increasing alcohol series (50%—70%—90%—95%—2 × 100%) before clearing them in a benzyl-mixture (two parts of benzyl benzoate: one part of benzyl alcohol). Brain slices were immediately mounted between coverslips in VECTASHIELD® Mounting Medium (Vector Labs).

The mouse α-TH antibody was also used in other preparations for the main labeling with the addition of phalloidin for the background. After fixation and washes, the specimen was incubated for 48 h in a 1:100 dilution of the mouse monoclonal α-TH antibody. After various rinses, the brain was incubated for 24 h in a 1:100 dilution of a DyLight 649 α-mouse (Jackson ImmunoResearch) and prepared as described above.

### Confocal Microscopy

Samples were imaged using a confocal laser scanning microscopy (Leica TCS SP5 MP and LSM510 NLO—Carl Zeiss, Jena, Germany), with either a 25× oil objective (LCI Plan-Neofluar 25×/0.8) or a 20× water objective (PL APO 20×/0.5 on the Leica microscope and W Plan Apo 20×/1.0 on the Zeiss microscope). Ar-Kr and HeNe lasers were used to excite Alexa Fluor® 488, Texas Red® DyLight 649 at 488, 543 and 633 nm, respectively. The emission was detected with a 500–530, 560–615 and 650–680 nm bandpass filter, respectively. Cy3 was excited and detected using the same setting as Texas Red, as they possess similar dye properties and deliver, for our purpose, identical results.

The images were collected as Z stacks with a Z step size between 0.410 μm and 0.709 μm. A visual field of view was registered with a pixel resolution of either 512 × 512 or 1024 × 1024 pixels. Each region of interest was captured by moving the visual field of view over the entire region, resulting in a huge and detailed mosaic image. The stacks were rendered for 3D reconstruction with Imaris 7.7 (Bitplane, Zurich, Switzerland). At least five samples were compared for each neuronal cluster considered to confirm the neuroanatomical processes reported.

### Nomenclature

The different spatial axes of orientation used in this study follow the body axes of the honey bee. The nomenclature used for characterizing brain structures and pathways follows that proposed by the Insect Brain Name Working Group (Ito et al., 2014).

## Results

### Dopaminergic Cell Clusters

**Figure 1:**
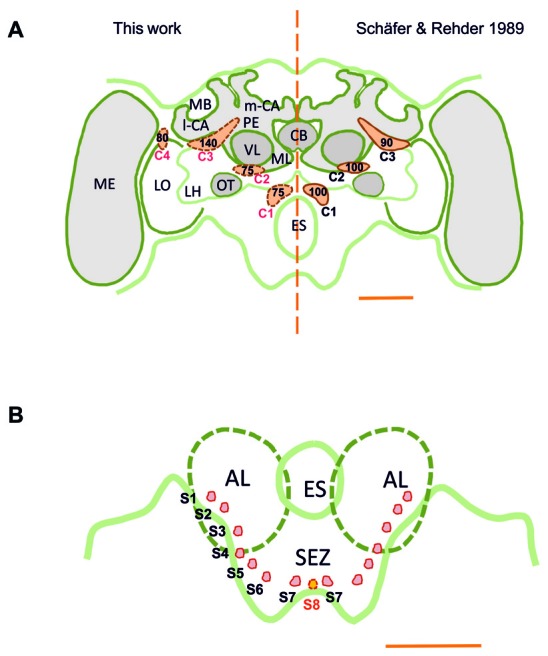
**(A)** Scheme of the honey bee brain in frontal view showing the main dopaminergic cell clusters, C1–C4 (in orange), found in the supraesophageal zone (SPZ); left: results of this study obtained using the technique of anti-TH labeling; right: results of Schäfer and Rehder (1989) obtained using dopamine-like immunoreactivity (DA-ir). The numbers indicate the numbers of neurons per cell cluster. The main difference resides in the identification of the C4 cluster, the existence of which was unknown until now. **(B)** Detail of the ventral brain region showing the AL and the SEZ of the brain. Small dopaminergic clusters S1–S7 are shown on each brain hemisphere. A novel dopaminergic cluster S8 is also shown. MB, mushroom body; m-CA, median calyx; l-CA, lateral calyx; PED, pedunculus; VL, vertical lobe; ML, medial lobe; CB, central body; ME, medulla; LO, lobula; OT, optic tubercle; LH, lateral horn; AL, antennal lobe; ES, esophagus; SEZ, subesophageal zone of the brain. Scale bar: 200 μm.

**Figure 2:**
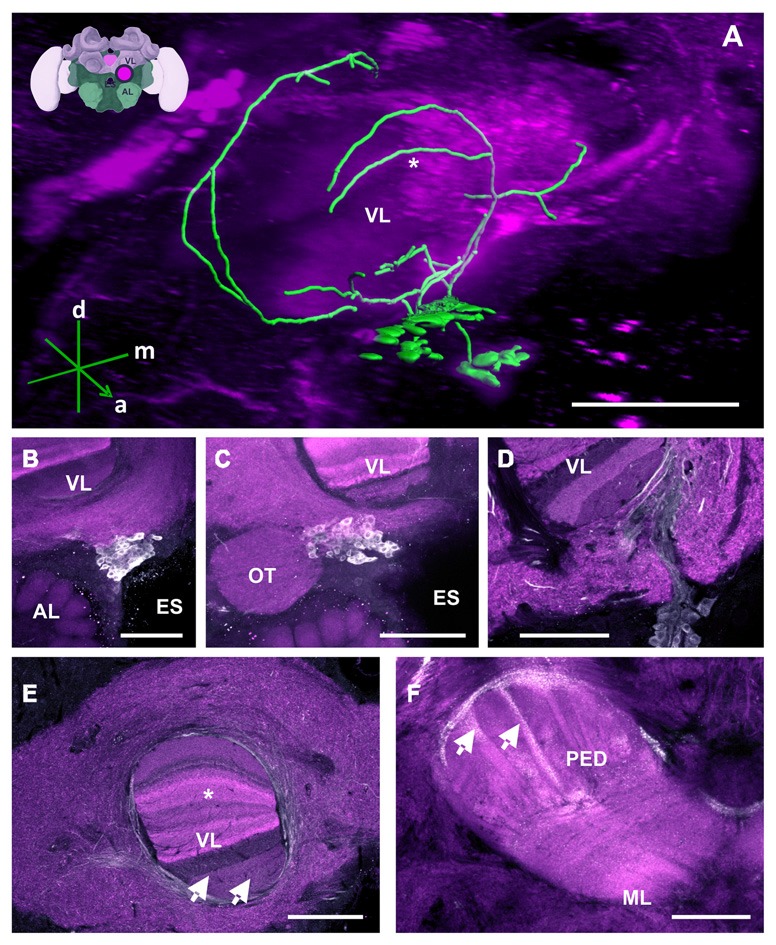
The C1 and C2 clusters and their processes in the bee brain. **(A)** 3D reconstruction of the C1 and C2 clusters. Confocal images of the C1 and C2 clusters were stacked and presented in an oblique angle, with reference axes indicating the directions of the anterior (a), dorsal (d) and medial (m) side of the stacks. The reconstructed neurons wrap the VL of the MB. The asterisk highlights an innervation of the VL described in panel **(E)**. Inset: localization (magenta spot) of the C1 and C2 clusters in a 3D reconstruction of the honey bee brain (adapted from Rybak et al., 2010). This reconstruction will be used in the following figures. AL, antennal lobe; VL, vertical lobe; ES, esophagus **(B)** The C1 cluster is located adjacent to the ES and between the VL of the MB and the AL. **(C)** The C2 cluster is located below the ventral part of the VL, above the AL and medial to the optic tuberculum (OT). **(D)** The neurite bundles of the C1 and C2 clusters meet at the ventromedial margin of the VL and then envelop and enter the VL. The lower layers of the VL can be clearly seen. **(E)** Fine fiber-like arborizations in the VL can be clearly observed in various layers of the VL (arrows). The neurite bundles envelop the VL dorsomedially and laterally. Parts of the dorsomedial bundle enter the VL at one of its most dorsal layers (asterisk—see panel **A** for the reconstruction), which presents Kenyon cell axons from the basal ring. Neurites arborize anteriorly to and out of the VL and make ramifications into the neuropils lateral and medial to the VL. **(F)** The PED of the MB is innervated by column-like varicosities (arrows). The main bundle can be traced back to processes running along the lateral border of the VL. ML, medial lobe, visible in this plane of section. Scale bar: 100 μm.

**Figure 3:**
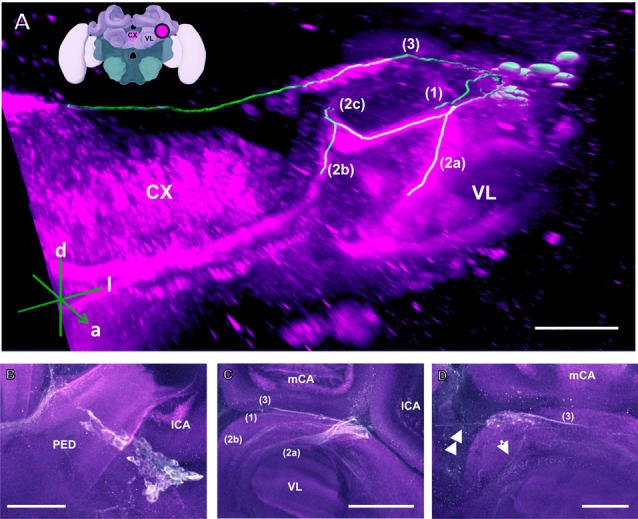
The C3 cluster and its main processes into the bee brain **(A)** 3D reconstruction of the C3 cluster and its main bundles (1; 2a, 2b, 2c; 3); confocal images of this cluster were stacked and presented in an oblique angle, with reference axes indicating the directions of the anterior (a), dorsal (d) and lateral (l) side of the stacks. The VL and the CX are shown. Inset: localization (magenta spot) of the C3 cluster in a reconstruction of the honey bee brain. CX, central complex; VL, vertical lobe. **(B)** The somata of the C3 cluster are located below the lateral calyces (lCA) of the MBs and adjacent to the PED. Various soma diameters can be observed. **(C)** Three main bundles can be traced from the C3 cluster: (1) the bundle projects to the upper division (UD) of the CX through the anterior part of the UD. (2) The bundle splits and sends one branch to the neuropil ventromedial to the VL (2a) and one branch to the contralateral side (2b); one branch (2c) projects posterior, along the dorsal rim of the VL, making a loop behind the PED (see Figure 5 for 2c). From between the lateral (lCA) and the medial calyx (mCA), the fibers invade the PED and the calyx neuropil (see Figure 5). (3) A bundle projects to an unidentified neuropil, flanking dorsally the UD of the CB and interconnects to the same region in the other brain hemisphere** (D)**. Innervations in a unidentified region ventral to the mCA. From this neuropil, one neurite bundle projects to the contralateral side (double arrows) and another one projects posteriorly (single arrow). Scale bar: 100 μm.

TH-ir was detectable throughout the whole brain, i.e., in the brain regions above and below the level of the esophagus (ES), the supraesophageal zone (SPZ) and the subesophageal zone (SEZ). Our results are consistent with previous reports of DA-immunoreactive labeling (Schürmann et al., 1989; Schäfer and Rehder, 1989), as we could identify three main dopaminergic clusters, C1–C3 (Figure 1A), in each brain hemisphere (Schürmann et al., 1989; Schäfer and Rehder, 1989). The C1 cluster is located in a small region adjacent to the ES and the antennal lobe (AL), at a depth of *ca*. 120 μm (see inset in Figure 2A). The C2 cluster is more eccentric (Figures 1, 2C) and situated above C1; it is located between the AL and the vertical lobe (VL), at a depth of *ca*. 60 μm. The C3 cluster is located below the calyces (CA) of the mushroom body (MB), from the ventral to the dorsal part of the brain (Figure 3).

In addition, we discovered a fourth cluster that we termed C4, which was overlooked in prior studies. This cluster is located above the dorsomedial border of the lobula (Figures 1, 7), spanning the anterior part of the brain down to a depth of *ca*. 120 μm. The discovery of this cluster contradicts prior statements mentioning that the Optic lobes (OLs) are devoid of DA labeling (Schäfer and Rehder, 1989). Several small clusters (S1–S7; Figures 1B, 11–14) were identified between the AL and the SEZ, including a novel dopaminergic cluster in the SEZ, which we termed S8 (Figures 1B, 14). Other smaller cell clusters were also detected: C3b (Figure 6) and S_p_ (Figure 8), which are two individual clusters with *ca*. 8 and 15–20 somata, respectively, located in each hemisphere around the protocerebral bridge (PB) and dorsal to the central complex (CX). Further dopaminergic clusters found are the anterior optic tubercle (AOTU) cluster, located below each anterior optic tubercle and presenting 2–3 somata (Figure 9) and the S_L_ cluster with its 5–8 somata at the border between the lobula and the deutocerebrum (Figure 10).

**Figure 4:**
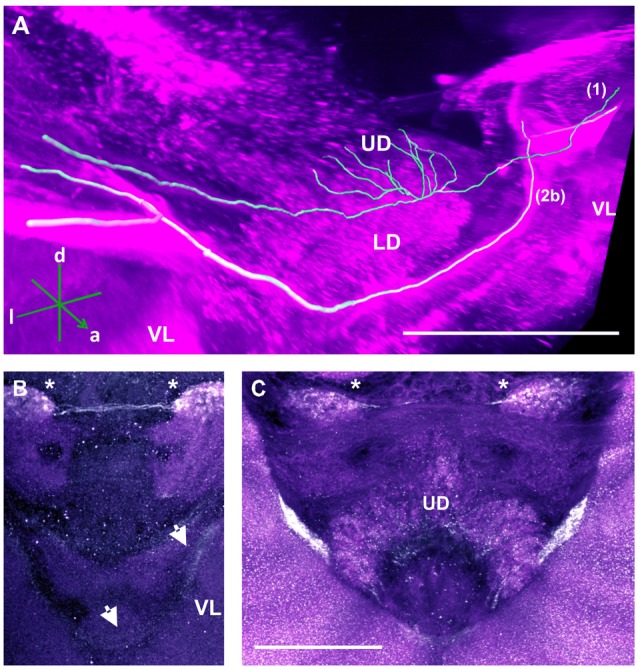
Two main tracts of the C3 cluster interconnecting with both brain hemispheres. **(A)** 3D reconstruction of these tracts; confocal images were stacked and presented in an oblique angle, with reference axes indicating the directions of the anterior (a), dorsal (d) and lateral (l) side of the stacks. Bundle 1 projects into the UD of the CX. Both VLs are indicated for reference. **(B)** A neurite bundle projects along the medial border of the VL of the MB to the anterior part of the brain, bypassing the CB and projecting further to the other brain hemisphere (arrows). The unidentified regions (asterisks) flanking the CB dorsally are interconnected via another bundle. **(C)** Another interconnecting neurite bundle projects to the anterior part of the UD of the CX before finally projecting further to the other brain hemisphere. Scale bar: 100 μm.

**Figure 5:**
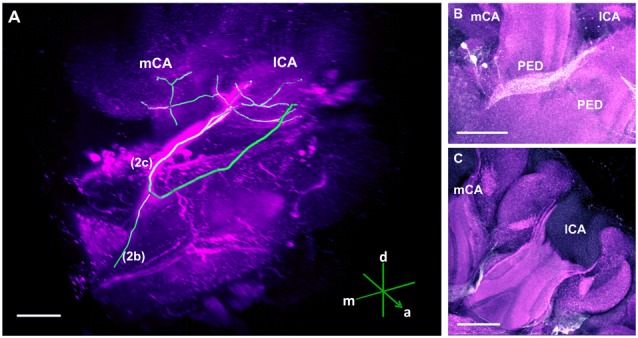
The main track of the C3 cluster innervating the calyces (CA) of the MB. **(A)** 3D reconstruction of this track; confocal images were stacked and presented in an oblique angle, with reference axes indicating the directions of the anterior (a), dorsal (d) and medial (m) side of the stacks. The mCA and the lCA calyces are shown. One neurite bundle originating from the C3 cluster projects to the contralateral hemisphere (2b) and another one forms a loop (2c) before terminating in the calyces of the MB. **(B)** The loop envelops the posterior part of the PED. It goes towards the space between the two pedunculi (PED) and projects into both calyces of the MB. **(C)** TH-ir in the calyces of the MB. Somata of the C3 cluster can be observed below one lCA. Scale bar: 100 μm.

**Figure 6:**
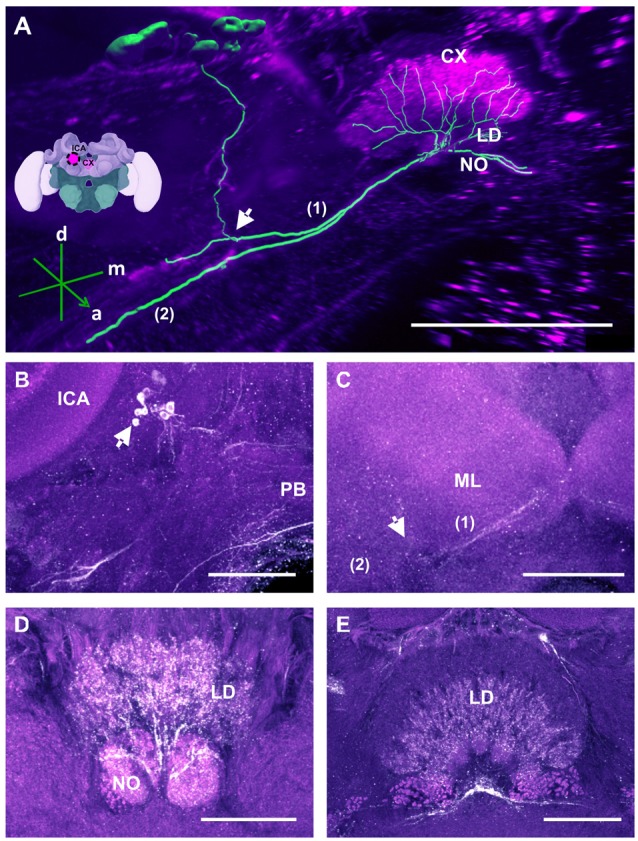
The C3b cluster and its processes in the CX. **(A)** 3D reconstruction of this cluster; confocal images of the C3b cluster were stacked and presented in an oblique angle, with reference axes indicating the directions of the anterior (a), dorsal (d) and medial (m) side of the stacks. The innervation of the CX is shown. One branch (1) projects medially toward the ventral border of the CB and enters the lower division (LD) and the noduli (NO). Another branch (2) goes to the ventral border of the ML. Inset: localization (magenta spot) of the C3b cluster in a reconstruction of the honey bee brain. CX, central complex; lCa, lateral calyx. **(B)** The cell cluster (arrow) consists of around eight somata located posterior to the PED and medioventrally to the lCA. The protocerebral bridge (PB) of the CX is shown. **(C)** From the somata, the neurites project ventrally (arrow; see also panel **A** arrow) where they divide into two branches, innervating (1) the CB, its NO and the LD of the CX—and (2) the ventral border of ML. **(D)** TH-ir in the NO and the posterior part of the LD. **(E)** TH-ir in the LD of the CX. The projections enter the CX ventrally and originate symmetrically from both C3b clusters. Scale bar: 100 μm.

**Figure 7:**
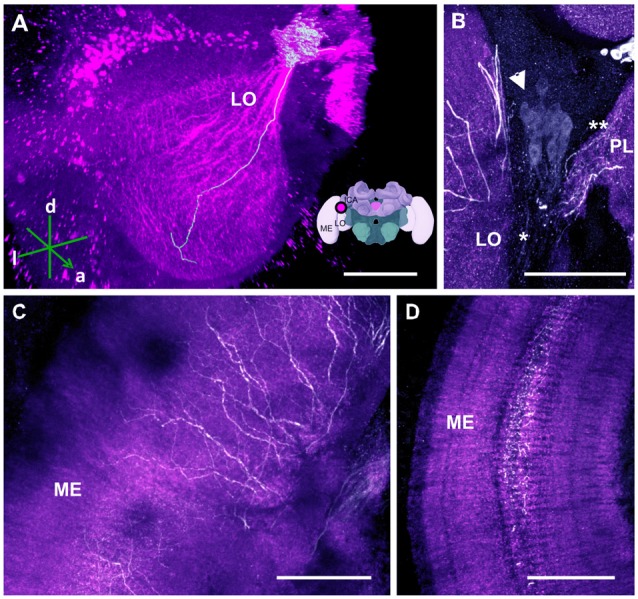
The C4 cluster and its projections to the visual neuropils. **(A)** 3D reconstruction of the C4 cluster and of a single traceable neurite projecting to the LO; confocal images were stacked and presented in an oblique angle, with reference axes indicating the directions of the anterior (a), dorsal (d) and lateral (l) side of the stacks. Inset: localization (magenta spot) of the C4 cluster in a reconstruction of the honey bee brain. ME, medulla; LO, lobula; lCa, lateral calyx. **(B)** Approximately 80 somata (arrow) are found in the C4 cluster. The neurite bundle (asterisk) projects toward the LO and the protocerebral lobe (PL; double asterisk). **(C)** Leaving the LO, the neurites arborize in the ME in a column-like pattern. **(D)** TH-ir in the serpentine layer of the ME. The neurites arborize laterally within the serpentine layer. Scale bar: 100 μm.

**Figure 8:**
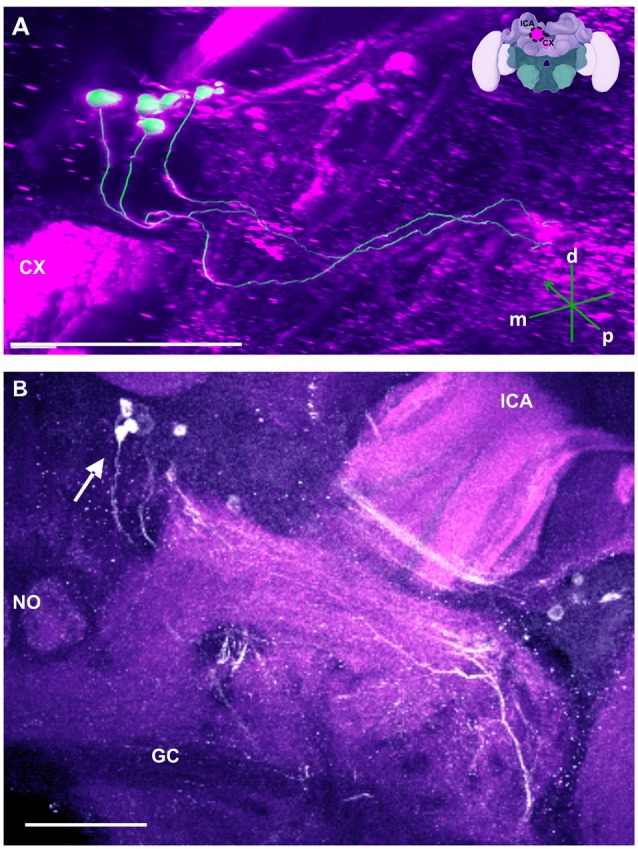
The S_P_ cluster and its neurite bundle. **(A)** 3D reconstruction of the S_P_ cluster; confocal images of this cluster were stacked and presented in an oblique angle, with reference axes indicating the directions of the posterior (p), dorsal (d) and medial (m) side of the stacks. CX, central complex. Inset: localization (magenta spot) of the S_P_ cluster in a reconstruction of the honey bee brain. lCA, lateral calyx. **(B)** Between 15 and 20 somata are found in this cluster (arrow). The neurite bundles form two main tracts projecting to neuropils dorsal to the great commisure (GC). The lCA and a nodulus (NO) are shown. Scale bar: 100 μm.

**Figure 9:**
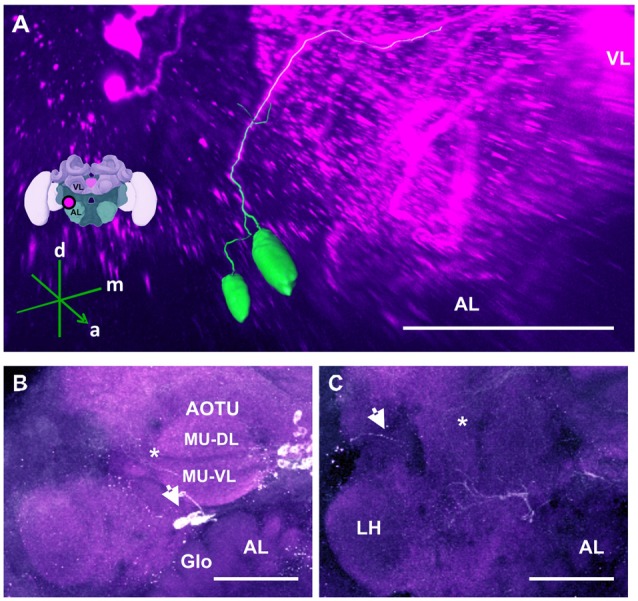
The anterior optic tubercle (AOTU) cluster and its neurite bundle. **(A)** 3D reconstruction of the AOTU cluster; confocal images of this cluster were stacked and presented in an oblique angle, with reference axes indicating the directions of the anterior (a), dorsal (d) and medial (m) side of the stacks. The location of the VL and the AL is indicated. Inset: localization (magenta spot) of the AOTU cluster in a reconstruction of the honey bee brain. **(B)** 2–3 somata (arrow) are located between the AOTU and the AL. Glomerular (Glo) structures can be seen in the AL. The two major units (MU) of the AOTU, the dorsal (MU-DL) and the ventral units (MU-VL), are shown. Processes leaving the AOTU can be observed, which are unrelated to the AOTU cluster (asterisk). **(C)** The neurite bundle projects posteriorly below the AOTU along the ventrolateral border of the VL, making a turn dorsomedially (asterisk) and finally projecting to neuropils posterior to the ML. Some processes toward the lateral border of the protocerebrum can be observed (arrow), superior to the LH. Their terminals could not be determined. AL, antennal lobe. Scale bar: 100 μm.

**Figure 10:**
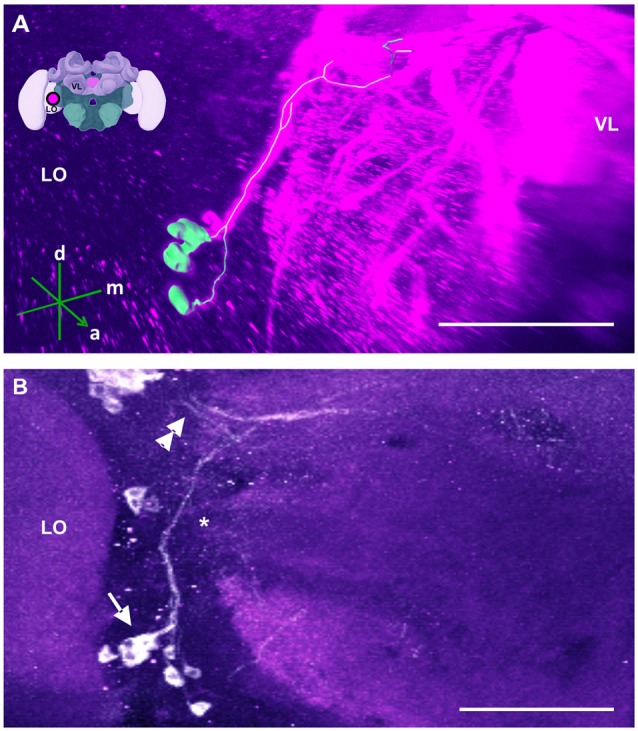
The S_L_ cluster and its neurite bundle. **(A)** 3D reconstruction of the S_L_ cluster; confocal images of this cluster were stacked and presented in an oblique angle, with reference axes indicating the directions of the anterior (a), dorsal (d) and medial (m) side of the stacks. The VL and the lobula (LO) are indicated. Inset: localization (magenta spot) of the S_L_ cluster in a reconstruction of the honey bee brain. **(B)** The S_L_ cluster with its 5–8 somata (arrow) is located in the ventromedial border of the LO. Its neurite bundle projects dorsally and turns medially (asterisk), where it collates with processes coming from the C4 cluster (double arrow). Their terminals could not be located. Scale bar: 100 μm.

**Figure 11:**
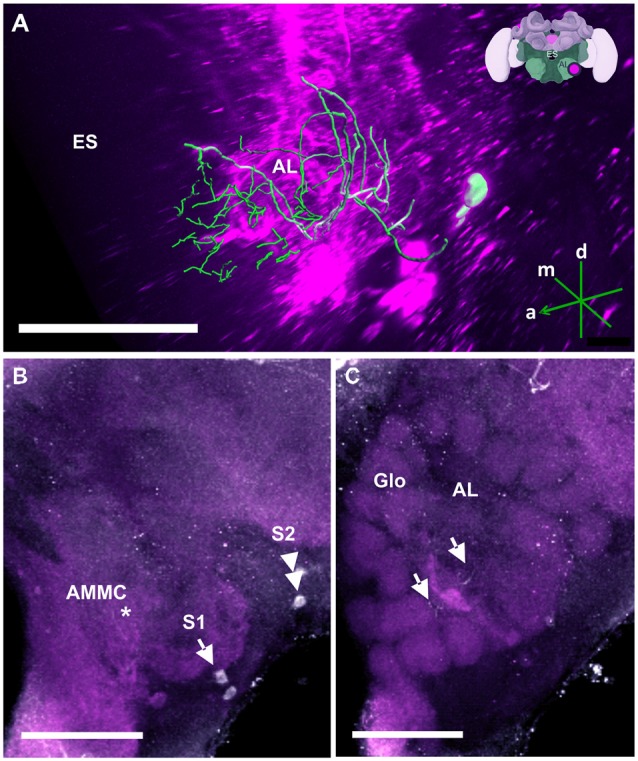
The S1 and S2 clusters and their processes in the AL. **(A)** 3D reconstruction of the S1 and S2 clusters; confocal images were stacked and presented in an oblique angle, with reference axes indicating the directions of the anterior (a), dorsal (d) and medial (m) side of the stacks. Location of the ES is indicated. Inset: localization (magenta spot) of the S1 and S2 clusters in a reconstruction of the honey bee brain (adapted from Rybak et al., 2010). **(B)** The somata of the S1 (arrow) and S2 clusters (double arrow) can be observed at the lateral border of the deutocerebrum. The neurites project medially to a neuropil in the antennal mechanosensory and motor center (AMMC) where they form fine arborizations (asterisk). **(C)** The neurite bundles form delicate arborizations innervating the AL in the center (arrows) and spread into the peripherally arranged Glo. Scale bar: 100 μm.

**Figure 12:**
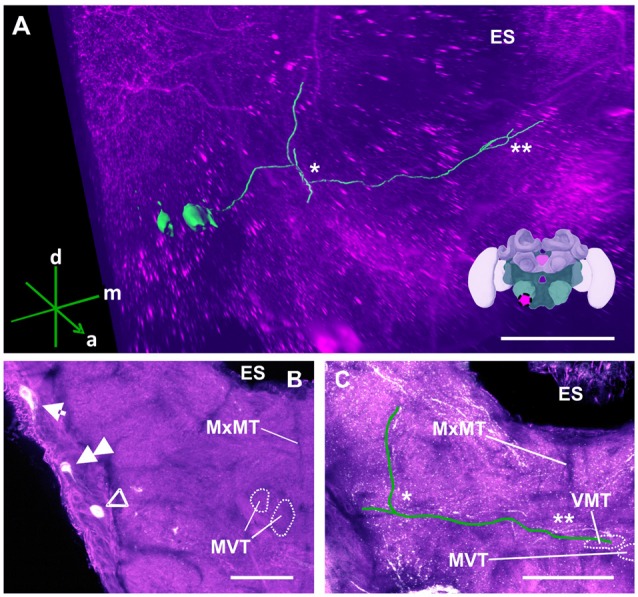
The S3 cluster and its processes. **(A)** 3D reconstruction of the S3 cluster; confocal images were stacked and presented in an oblique angle, with reference axes indicating the directions of the anterior (a), dorsal (d) and medial (m) side of the stacks. The first ascending branch to the AMMC and the location where the neurite crosses the midline are indicated by * and **, respectively. The location of ES is also indicated. Inset: localization (magenta spot) of the S3 cluster in a reconstruction of the honey bee brain. **(B)** The somata of the S3 cluster (arrow) can be observed at the lateral somatal rind of the SEZ. Additionally, the S4 (double arrow) and S5 clusters (open arrowhead) are shown. The appearance of the maxillary midline tract (MxMT) and the division of the median ventral tract (MVT) provide the approximate location of these clusters in the SEZ. ES, esophagus. **(C)** The main neurite of the S3 cluster is shown parallel to the green line to allow its tracing on the confocal projection among the extensive dopaminergic network in the SEZ. On the ipsilateral side, it ascends (asterisk) to the AMMC. It also crosses the midline (double asterisk). The terminal appears to end in the ventral median tract (VMT) and the MVT. The MxMT is still observable in this projection. ES, esophagus. Scale bar: 100 μm.

**Figure 13:**
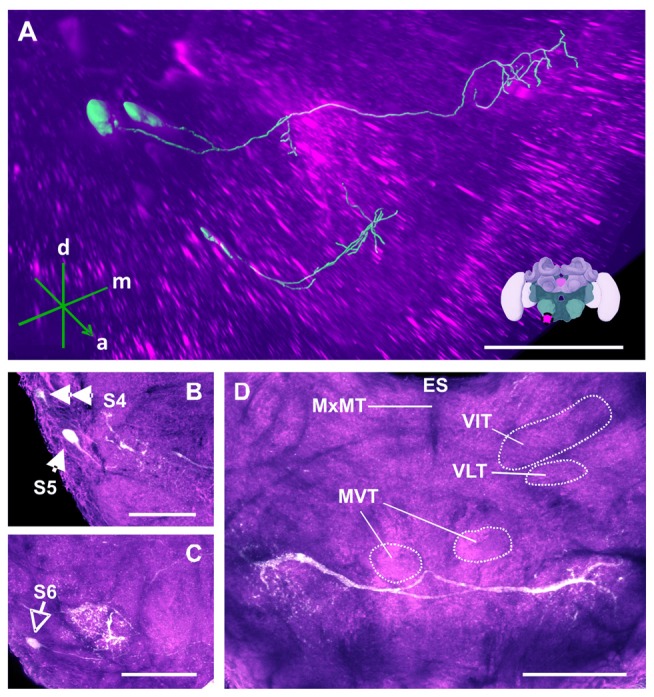
The S4, S5 and S6 clusters and their processes. **(A)** 3D reconstruction of both clusters; confocal images were stacked and presented in an oblique angle, with reference axes indicating the directions of the anterior (a), dorsal (d) and medial (m) side of the stacks. Inset: localization (magenta spot) of the S5 and S6 clusters in a reconstruction of the honey bee brain. **(B)** The somata of the S5 cluster (arrow) can be observed at the lateral border of SEZ. The S4 cluster (double arrow) can also be observed. **(C)** The somata of the S6 cluster (empty arrowhead) can be seen at the lateral border of SEZ. The main neurites innervate a proximate region on the ipsilateral side. **(D)** The main neurite bundle of the S5 cluster crosses the midline and innervates the contralateral side. Several prominent tracts in the SEZ can be observed, such as the MxMT, the ventral intermediate tract (VIT), the ventral lateral tract (VLT) and the MVT. ES, esophagus. Scale bar: 100 μm.

**Figure 14:**
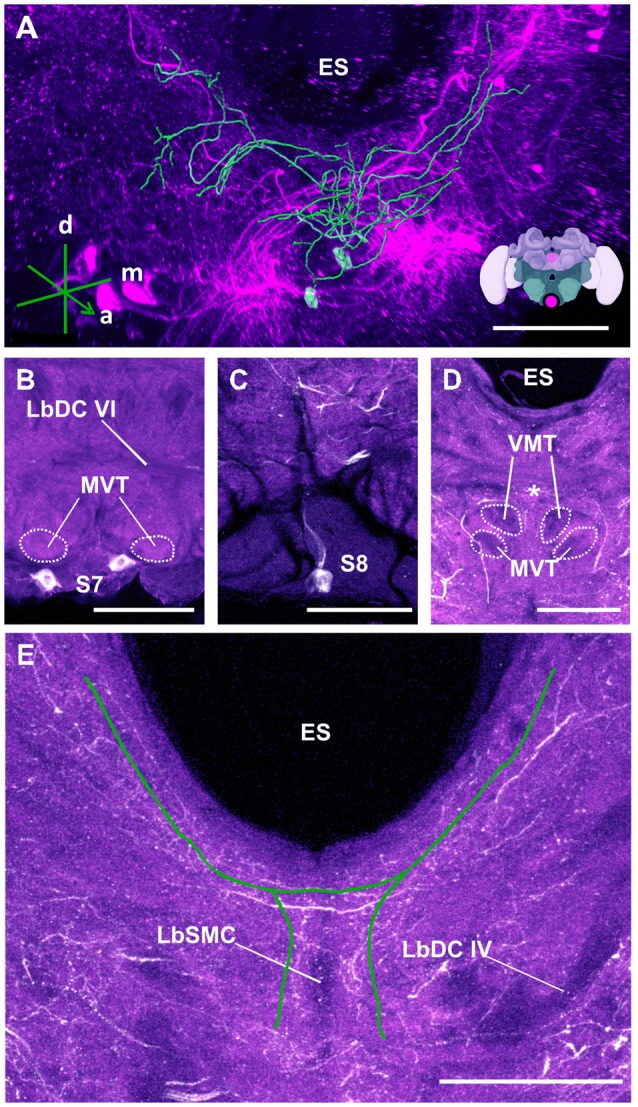
The S7 and S8 clusters and their processes. **(A)** 3D reconstruction of these clusters; confocal images were stacked and presented in an oblique angle, with reference axes indicating the directions of the anterior (a), dorsal (d) and medial (m) side of the stacks. ES, esophagus. Inset: localization (magenta spot) of the S7 and S8 clusters in a reconstruction of the honey bee brain. **(B)** The somata of the S7 cluster can be observed inferior to the MVT on the ventral somatal rind of the SEZ. LbDC VI, labial dorsal commissure VI. **(C)** The somata of the S8 cluster can be seen at the ventral border of the SEZ. The cluster sends its main neurite along the midline of the brain. **(D)** The neurite bundles from the S7 and S8 clusters envelop the VMT and the MVT. Their signals collapse at the midline (asterisk). **(E)** An important part of the neurite bundles abundantly innervate neuropils located along the proximate border of the ES. LbSMC, labial superior median commissure; LbDC IV, labial dorsal commissure IV. Scale bar: 100 μm.

### Dopaminergic Cell Numbers

Our counting of dopaminergic neurons (Figure 1) in the C1 and C2 clusters yielded around 75 somata per cluster; less than the 100 somata (Schäfer and Rehder, 1989) and more than the 40 somata (Schürmann et al., 1989) previously reported for these clusters. Each soma had a diameter of *ca*. 10 μm, similar to the size previously reported. In the C3 cluster, we identified *ca*. 140 somata, which is more than the 80–90 somata (Schäfer and Rehder, 1989) and 50 somata (Schürmann et al., 1989) previously reported. The somata within this cluster had diameters varying between 7 μm and 12 μm. In the C4 cluster, which had not been previously described, *ca*. 80 somata were identified. Their diameters ranged between 8 μm and 10 μm.

Taking into account the various smaller clusters mentioned in the previous section, we counted a total of 400–450 somata per brain hemisphere (range of several samples), an estimation that surpasses the 350 and 120 somata reported by Schäfer and Rehder (1989) and Schürmann et al. (1989), respectively. TH-immunoreactive clusters and their processes were located symmetrically within both brain hemispheres. Signal intensity of the newly identified C4 cluster showed notable variability. It varied across samples and was less robust compared to that of other clusters, a fact that may explain why it was overlooked previously.

### Dopaminergic Innervation of Brain Regions

#### Dopaminergic Innervation in the Supraesophageal Zone (SPZ)

##### Mushroom bodies (MBs)

MBs are prominent higher-order integration centers, which receive input from olfactory, visual, gustatory and mechanosensory afferents and from the lateral protocerebrum (LP; Strausfeld, 2002). Each MB is made of approximately 170,000 Kenyon cells (Witthöft, 1967) and has a pair of cup-like neuropils called the calyces, one of which is located in the medial zone and the other in the lateral zone of the brain. Both calyces are connected to a common Pedunculus (PED), which divides into a medial and VL. The VL extends forward to the front surface of the brain where it truncates and lies approximately at 150 μm above the AL. The cell bodies of the Kenyon cells (class I Kenyon cells) are located in the bowl of each calyx and above its rim. Their dendrites ramify within the cup-shaped neuropil of calyces while their projection fibers pass through the PED, branch at its base, and send one process into the medial lobe (ML) and another process into the VL. An additional group of cell bodies lies outside each calyx (class II Kenyon cells) and forms a layer around the outer calyx wall. The neurites of these cells penetrate the outer wall to project directly towards the lower part of the VL, which has been identified as the gamma lobe (Strausfeld, 2002).

TH-ir showed that the MB is innervated by the three main clusters C1, C2 and C3, at the level of the vertical and ML, the PED, and the calyces (Figures 2–5). Neither the Kenyon cells nor any MB intrinsic neurons were labeled. We have divided our description of MB innervation according to the three main regions of this structure: the lobes, the PED and the calyces.

###### Medial and vertical lobes.

The neurites of the C1 and C2 clusters meet at a point posterior to each cluster (Figure 2D). Before meeting, they arborize laterally with intense immunolabeling around the outer medial and lower border of the VL, innervating various regions of the LP (Figure 2E). The TH-ir in the MB seems to come from the same bundle of neurites and can be detected in both the vertical and the MLs. In the VL, innervations comprising various layers are observed, with a higher intensity found in the inferior region, corresponding, in part, to the gamma lobe (Figure 2E; Strausfeld, 2002). These projections cannot be distinctly traced as they appear as thin fiber-like arborizations. In the ML, faint and variable signals can be detected, indicating the presence of very fine dopaminergic branches.

The C3 cluster sends one of its neuritic bundles in the direction of the midline of the brain. It splits into three main branches (Figures 3A,C; branches termed 2a, 2b and 2c). One branch runs anteriorly and splits medially and laterally at the border of the VL. It projects further ventrally and appears to envelop the VL at its outer dorsal border (Figures 3A,C; branch 2a); another branch continues medioventrally to the midline sending projections to the contralateral hemisphere (Figures 3A,C; branch 2b). The last branch projects posteriorly along the medial surface of the VL and turns dorsolaterally behind the PED of the medial calyx (mCA; Figures 3A, 5A; branch 2c). This thick branch terminates between the medial and lateral calyces (lCA; Figure 5B). Furthermore, the lateral projection of the bundle coming from the C3 cluster runs anteriorly where it branches to innervate the PL and then runs further ventrally along the border of the VL. All these branches show strong anti-TH labeling.

###### Pedunculus (PED).

TH-ir originating from the layers of the VL continues further posteriorly and dorsally as fine fibers projecting into the PED where they disperse into columns built by Kenyon cell axons (Figure 2F, arrows). An intensely labeled bundle runs along the ventral and lateral border of the VL. It innervates the PED as a net of varicosities at the level where the PED starts to diffuse into the VL. Its origin can be located at the ventral border of this lobe where it diverges from a group of several large processes. Due to the inter-tangled nature of these processes, the neurites could not be traced further. The pedunculus neck (PEDN) is innervated by processes terminating as fine varicosities between the lateral and medial calyces (Figure 2F).

###### Calyces.

In the calyces, TH-ir exhibits an heterogeneous distribution (Figure 5C). The lip and the collar, which receive olfactory and visual input, respectively (Gronenberg, 1999; Ehmer and Gronenberg, 2002; Strausfeld, 2002), present indistinct varicose arborizations whilst the basal ring, which receives olfactory and visual input (Gronenberg, 2001), provides comparatively weaker signals. Unfortunately, it was not possible to determine the location of the somata connected to these arborizations.

##### Central complex (CX)

TH-ir in the CX revealed that dopaminergic processes could be traced back to at least two origins. The first one contributes to the projections in the anterior part of the upper division (UD) of the CB. It derives from the large neurite bundles coming from the C3 cluster (Figures 3A,C; bundle 1). One of these bundles contacts a small region in the PL located superior to the VL and anterior to the PED. There it gives rise to numerous varicosities. Afterwards it projects ventromedially to the midline where it innervates the UD of the CB in the form of densely packed column-like arborizations, invading it in a compartment-wise manner (Figure 4; bundle 1). The second bundle contributes to the projections in the posterior part of the UD, the lower division (LD) of the CB, and the noduli (NO). It can be traced back to a set of *ca*. eight labeled somata in each hemisphere that are positioned in a row posterior and lateral to the PB and dorsolaterally to the l-CA (Figure 6B, arrow). We call this cluster C3b (Figure 6), because its somata also arborize into the CX, similarly to neurons of the C3 cluster. Figure 6A shows a 3D reconstruction of the C3b neurons to provide a fuller characterization of their morphology. Their neurites run ventrally and project anteriorly towards the ventral border of the ML (Figure 6C). There, the neurites send one branch medially towards the ventral border of the CB from which the labeled fibers enter the lower and UDs of the CB and the NO (Figures 6A,C, arrow). The posterior part of the UD is also innervated at its posterior surface by a number of thin fibers. The PB shows only weak labeling. In general, the intensity of TH-ir in the posterior part of the CX is stronger compared to the anterior part (Figures 4C, 6D,E).

##### Optic lobes (OLs)

The OLs are responsible for processing visual information acquired via the photoreceptors located within the ommatidia of the compound eyes (Avargues-Weber et al., 2012). They comprise three main regions: the lamina, medulla and lobula. Previous studies using DA-ir did not find dopaminergic innervation in these brain regions (Schürmann et al., 1989; Schäfer and Rehder, 1989). Using anti-TH labeling, however, we detected dopaminergic processes in these neuropils, which were derived from a single cluster located at the dorsomedial border of the lobula (i.e., the C4 cluster; Figures 1, 7A,B). This cluster has neurites sending processes both to the OLs (Figure 7B, asterisk) and the PL at the level of the PEDN where the terminals could not be detected (Figure 7B, double asterisk).

Two TH antibodies yielded different labeling results at the level of the OLs. Immunolabeling with the TH antiserum raised in rabbit exhibited two subtypes of projections. One subtype consisted of two projections that bypassed the lobula and ran along the dorso- and ventroanterior border of the OL before innervating the medulla. Each bundle comprised large neurites that were intensely labeled and innervated the outer layer of the medulla. They seemed to share the same projection tract running along the dorsal and ventral border of the OL, the anterior superior optic tract (asot), and the anterior inferior optic tract (aiot; Ehmer and Gronenberg, 2002). The projections of the second subtype formed a fan-shaped bundle of relatively large neurites that projected ventrally and entered laterally into the lobula. They innervated this neuropil homogenously at different depths. Intense labeling was also detected in the medulla’s serpentine layer (Figure 7C) and in column-like processes of the medulla (Figure 7D). Moving towards the outer layer of the medulla, the neurites of the C4 cluster appeared in the form of column-like thin fibers.

Immunolabeling with the TH antibody raised in mouse uncovered only the second projection subtype. Additionally, both antibodies labeled the retina. We therefore reconstructed only the second subtype as its somata were traceable and could be detected by the two antibodies. Our 3D reconstruction was able to trace a process that projected uninterrupted to the lobula (Figure 7A).

##### Other neuropils in the protocerebral lobe

Our anti-TH labeling revealed another projection, which could be traced back to the C3 cluster, and which was not detected in previous reports (Schürmann et al., 1989; Schäfer and Rehder, 1989). This projection reached a small region, which was located posterior to the anterior dorsal protocerebral commissure (adpc) and flanked (dorsally) the UD of the central body (CB; Figures 3D, 4B,C, asterisks). The innervations were strong and bleb-like in comparison with the size of its neurites. Two other faint projections were present in this neuropil. The first one (Figure 3D double arrows) reached the contralateral hemisphere. The second one (Figure 3D, arrow) left posteriorly and turned dorsoposteriorly around the PEDN, where it intertwined with other processes (for example, those originating from the C4 cluster) thus rendering its terminals in the PL untraceable.

Besides this novel projection, some TH-immunoreactive processes were similar to those described before (Schürmann et al., 1989; Schäfer and Rehder, 1989). For example, posterior to the neuropil mentioned above (see Figure 3D) and dorsal to the mCA, and anterior to the PB, there are few somata (Figure 8B, arrow) that project ventrally crossing the neurite of the C3b cluster. This cluster has been termed the S_P_ cluster (Schäfer and Rehder, 1989). Its projections continued to a neuropil that was dorsal to the great commissure (GC) where they sent very thin side processes into the neuropil at the lateral border of the PB (Figure 8). Figure 8A shows a 3D reconstruction of this cluster and its processes.

The AOTU are small neuropils located in each hemisphere of the insect brain, which are connected by two inter-tubercle tracts (Mota et al., 2011b). They are a major target of visual interneurons from the OL, in particular, from the lobula and the medulla (Mota et al., 2011b). They respond to chromatic information in a spatially and temporally segregated manner and are thought to participate in navigation (Mota et al., 2013). We found that the AOTUs are innervated by dopaminergic varicose processes, which, at least in part, can be traced back to a set of about five labeled fibers that run in the inter-tubercle tracts. Due to its location adjacent to the AOTU, this cluster is called the AOTU cluster (Figure 9). The location of its somata, however, could not be determined (Figure 9B, asterisk).

Below each AOTU, 2–3 somata (Figure 9B) with a diameter of 20–25 μm projected posteriorly to the ventrolateral border of the VL of the MB, ascending and making widespread arborizations in the neuropil lateral to the PED (Figure 9C, star). A few of their processes projected towards the lobula (Figure 9C, arrow), but did not enter the OL.

A further cluster named S_L_ was located at the ventroposterior border of the lobula (Schäfer and Rehder, 1989; Figure 10A). It consisted of 5–8 somata (Figure 10B, arrow) and gave rise to a thin bundle of neurites that projected dorsally and made a medial turn before reaching the l-CA of the MB. Some of the processes remained in the vicinity of the calyces, while others projected behind the CB across the midline of the brain. The terminals of these fibers could not be detected.

##### Antennal lobes (AL) and antennal mechanosensory and motor centers (AMMC)

In the AL (Figure 11), TH-ir could be detected in two small clusters of neurons termed S1 (Figure 11B, single arrow) and S2 (Figure 11B, double arrow), which were located in the SEZ. Each cluster contained two somata with a diameter of 10–20 μm. Both were located in the immediate region posterior to the AL, at the lateral border of the AMMC, with S1 being more ventral and anterior than S2. Their projections shared a similar morphological pattern. Tracing their resolution to the single-cell level was difficult despite their relatively large sizes (Figure 11A). The neurites projected medially to a neuropil in the AMMC where they formed delicate arborizations (Figure 11B, asterisk) before entering the AL. The branches were distributed as fine processes all over the AL, making contacts with fibers across glomeruli (Glo) of the AL (Figure 11C, arrows).

#### Dopaminergic Innervation in the Subesophageal Zone (SEZ)

TH-ir in the SEZ showed an extensive network of labeled fibers with their projections overlapping some of these tracts. Despite some minor differences in arborizations, we confirmed the presence of the previously reported dopaminergic clusters S3–S7, which were found in the ventral (S3–S6) and lateral (S7) somatal rind of each SEZ hemisphere. Furthermore, we discovered a new S8 cluster, which was located in the lateral somatal region. In total, we identified the presence of eight paired neurons within each SEZ hemisphere (S3–S7) and two unpaired neurons (S8).

The S3 cluster (Figure 12) contained two somata with a diameter between 15 μm and 20 μm (Figure 12B, arrow). The neurites of this cluster innervated different regions of the ipsi- and contralateral sides (Figure 12A). The main arborizations of the S3 neurons remained ventral to those of the AL neurons S1 and S2 (see above). After leaving the somatal rind, the neurites of the S3 cluster projected ipsilaterally to the antennal mechanosensory and motor center (AMMC; Figures 12A,C, asterisk). Before reaching it, however, the signals were mixed with those coming from the S7 and S8 cluster (Figure 14), rendering them indistinguishable. The main neurites continued to cross the SEZ midline (Figures 12A,C, double asterisk), possibly innervating both the medial ventral tract (MVT) and the ventral medial tract (VMT). This innervation pattern could not be clarified, as there was no clear distinction between these signals and those from the S7 and S8 clusters (Figure 14E).

The somata of the S4 cluster were located in the lateral somatal rind of the mandibular neuromere. This cluster was previously reported to contain 6–8 DA-immunoreactive somata with diameters between 8 μm and 11 μm (Schäfer and Rehder, 1989). In our case, we were able to detect only two labeled somata of around 10–15 μm (Figure 12B, double arrow), which probably correspond to the two neurons of this cluster. Although Schäfer and Rehder (1989) traced the neurites of these somata, within the SEZ neuropil, our anti-TH labeling yielded faint signals (Figure 12B) that disappeared in the extensive network of other labeled fibers.

The S5 cluster (Figure 13) was reported to contain two somata, 18–20 μm in diameter, arranged in two bilateral pairs that send their major projections through a labial ventral commissure into the contralateral hemiganglion (Schäfer and Rehder, 1989). Our labeling also showed the presence of two somata, 15–20 μm in diameter, within each SEZ hemisphere. Figure 13A shows a 3D-reconstruction of the S5 cluster with its main neurites having bilateral innervation of the SEZ hemispheres. They projected through the labial ventral commissure II (LbVC II) into the contralateral identical region, and also ipsilaterally (Figure 13D). The dendrites of the contralateral side were more prominent and bleb-like while the dendrites of the ipsilateral side were more fiber-like. Although the two types of dendrites innervate the same region and occasionally intertwine, their innervation pattern occurs within different depths without any observable overlap.

The S6 cluster (Figure 13) was previously described as consisting of two somata, 20–24 μm in diameter, located in the lateral somatal rind of the labial neuromere (Schäfer and Rehder, 1989). Our labeling identified the same two somata; unlike those of neurons in the S3, S4, S5 and S7 clusters, the processes of these somata did not decussate, but remained restricted to the ipsilateral half of the SEZ (Figure 13A). The projections of the S6 cluster descended ventrally and innervated the same region as the S5 cluster (Figure 13C), wherein they created a dopaminergic network.

The S7 cluster (Figure 14) was described as containing two bilaterally arranged somata, 24–30 μm in diameter, in the ventral somatal rind (Schäfer and Rehder, 1989). We also located these two somata, 20–25 μm in diameter (Figure 14B) and identified their neurites, which ascended in a tract lateral to the labial midline tract and branched in the dorsal neuropil of the SEZ (Figure 14B).

Finally, the S8 (Figure 14) cluster is reported here for the first time. It consisted of a pair of medially-located unpaired neurons with a diameter *ca*. 15–20 μm (Figure 14C). The S8 cluster sent its projection dorsally over the labial midline tract (Figure 14C). The neurites of this cluster intertwined with those of the S7 cluster close to their somata, making it impossible to delineate their projections (Figure 14A). Together, the neurites of the S7 and S8 clusters envelop the MVT and the VMT (Figure 14D). At the point where the labeling of both clusters became inseparable, some branches projected posteriorly and formed a bridge while others appeared to cross over dorsally and continue to ascend to the ventral border of the ES, where they further bifurcated and innervated regions along the ES up to the AMMC (Figure 14E).

## Discussion

In this study, we characterized the distribution pattern of dopaminergic neurons in the central nervous system of the honey bee using TH-ir. Two different commercially available TH antibodies were used, one a polyclonal raised in rabbit and the other a monoclonal raised in mouse; similar results were obtained with each antiserum apart from the expression pattern in the OLs. Our methods also yielded results partially similar to those previously reported (Schürmann et al., 1989; Schäfer and Rehder, 1989), wherein three main dopaminergic clusters, C1–C3 (Figure 1), were identified. Some minor clusters previously identified (Schürmann et al., 1989; Schäfer and Rehder, 1989) were also observed. Not previously reported, however, was a novel cluster, C4, located above the dorsomedial border of the lobula, which innervated the visual neuropils of the bee brain (Figures 1, 7). A novel eighth cluster, S8, in the ventral somatal rind of the SEZ was also detected for the first time.

Differences inherent to the labeling techniques employed in the prior and present study could account for the discovery of novel dopaminergic clusters. However, at least three studies in insects have shown that the labeling patterns obtained with DA and TH antibodies are not different (Nässel and Elekes, 1992; Hörner et al., 1995; Hamanaka et al., 2016). This suggests that both antisera recognize the same sets of “dopaminergic” neurons. Thus, a likely explanation for the differences between the present and previous studies resides in the fact that 12 μm thick wax sections (which require heating to >50°C) and a conventional light microscope were used in prior studies (Schürmann et al., 1989; Schäfer and Rehder, 1989), while thicker sections (80–160 μm) and confocal and confocal microscopy were used in our work. Given this thickness difference, lower values for cell counts in our study could be hardly attributed to tissue damage or loss during the sectioning process.

### The C1, C2 and C3 Clusters

The location and general connectivity of the C1–C3 clusters were consistent with those reported previously (Schürmann et al., 1989; Schäfer and Rehder, 1989). There were, however, slight differences, such as the number of somata and an unreported projection in the small neuropil flanking (dorsally) the CX.

**Figure 15:**
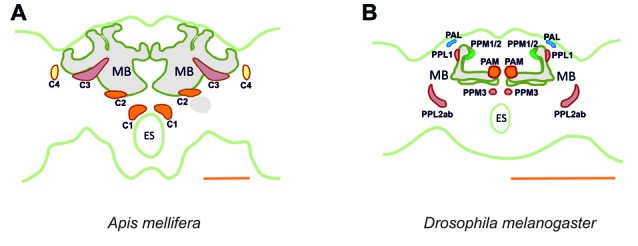
Main dopaminergic clusters known in the honey bee brain **(A)** and in the fruit fly brain **(B)**. Similar colors between both panels are used to suggest potential correspondences between dopaminergic clusters in the two species. In the bee, clusters C1, C2 are shown in orange, C3 in red and C4 in yellow. In the fly, the clusters shown are the protocerebral anterior medial (PAM) cluster, in orange, the protocerebral posterior medial (PPM) clusters 1/2, in green and the protocerebral posterior medial cluster 3 (PPM3), in red, the protocerebral posterior lateral (PPL) clusters 1 and 2ab, in red, and the protocerebral anterior lateral (PAL) cluster in blue. MB, mushroom body; ES, esophagus. Scale bar: 200 μm.

It is important to link the identities of other previously published cell profiles in the honey bee brain to those that are most likely to be dopaminergic. For example, neurons we identified in the C1 and C2 clusters resemble the A1 and A2 MB extrinsic neurons (MBEN) previously described (Rybak and Menzel, 1993). The A1 and A2 MBENs are located anteriorly and in the same depth as the C1 and C2 clusters, projecting in the same manner into the VL of the MB. Their branches envelop the VL and project to the PL unilaterally. Fine varicose fibers are detected in both the vertical and the ML. Rybak and Menzel (1993) counted an average of 50–60 labeled neurons of the A1 and A2 type. This number is close to the 75 somata counted both in the C1 and C2 clusters, thus indicating that these clusters may comprise these two types of MBENs. Possibly, the A1 and A2 neurons described by Rybak and Menzel as MBEN may in fact be the dopaminergic neurons of the C1 and C2 clusters. In *Drosophila*, the homolog of the C1 and C2 clusters may be the protocerebral anterior medial (PAM) cluster with respect to soma location and innervation pattern (Figure 15). Neurons within the PAM cluster also terminate in the MLs of the MB and in neuropils adjacent to them (Mao and Davis, 2009).

Neurons in the C3 cluster resemble the A6 MBENs reported by Rybak and Menzel (1993). Even though neurons in the C3 cluster could not be fully traced, it is possible to sort out a part of their massive projections by way of what is known about the A6 MBEN. The somata of the A6 MBENs are located ventrally to the l-CA of the MB and project to the VL, to neuropils in the contralateral hemisphere, and to the ipsilateral lateral horn (LH). Looking at the number of reported A6 neurons, which ranges between 60 and 80, we can reasonably conclude that they are part of the 140 cells comprising the C3 cluster. The homologs of this cluster in *Drosophila* are likely the protocerebral posterior lateral (PPL) clusters 1 (PPL1) and 2ab (PPL2ab), but also the protocerebral posterior medial cluster 3 (PPM3; Figure 15). First, the somata of the PPL1 cluster are relatively close to the calyx and have terminals in the dorsal part for the fan-shaped body as one of the terminals of the C3 cluster (Mao and Davis, 2009; Liu Q. et al., 2012). Second, the terminals of the PPL2ab cluster are detectable in the calyx of the MB (Mao and Davis, 2009) similar to the C3 cluster. Third, the PPM3 cluster arborizes into the CX like the bee C3 and C3b clusters (Mao and Davis, 2009). It is notable that C3 cluster is big, consisting of different types of neurons or, possibly, sub-clusters.

### The C4 Cluster

Our anti-TH labeling uncovered the presence of the C4 cluster, which innervated the OL of the honey bee. In accordance to the nomenclature of previous reports, the name adopted for this cluster follows the sequential order of the main dopaminergic clusters previously described in the honey bee brain. It is somewhat surprising that the C4 cluster has remained undetected in two parallel neuroanatomical characterizations of dopaminergic neurons (Schürmann et al., 1989; Schäfer and Rehder, 1989), even though Mercer et al. (1983) had first reported faint dopaminergic expression in the OLs. Despite attaining variable levels of DA expression, subsequent reports have confirmed such DA labeling (Taylor et al., 1992; Sasaki and Nagao, 2001).

In worker bees, regardless of their age, the labeling of DA in the OLs is relatively low when compared to that in the protocerebrum (Mercer et al., 1983; Sasaki and Nagao, 2001). An age/caste effect exists and DA levels are higher in bee foragers (Taylor et al., 1992). One cannot rule out, therefore, that in prior studies relatively young bees were used that had levels of DA expression that were below detectability.

The C4 cell morphology partially resembles that of the MBENs that project to the calyces and the OLs (Ehmer and Gronenberg, 2002). The C4 cluster may also contain at least three different cell types: (1) the first one shares the track with the asot along the dorsal border of the OL; (2) the second one shares the track with the aiot along the ventral border; and (3) the third one projects directly into the lobula and then innervates the serpentine layer of the medulla. These three types of neurons share only one single projection into the protocerebrum: it runs to the PEDN where the neurites could not be distinguished from other putative dopaminergic processes.

Even though these neurons share several properties of the MBENs mentioned in prior studies, it is intriguing to see that the cluster is located exclusively at the dorsomedial border of the lobula. Neurons projecting to the asot and aiot have their somata in the dorsomedial edge of the medulla and the base of the l-CA adjacent to the ventral edge of the medulla and lobula, respectively (Ehmer and Gronenberg, 2002). C4 cluster somata cannot be located in these regions, however, suggesting that they might possess different morphological properties. A corresponding dopaminergic cluster in *Drosophila* cannot be identified. In the fly, the protocerebral anterior lateral (PAL) cluster, which is lateral to the dorsal portion of the VLs (Mao and Davis, 2009), extends its processes to innervate the contralateral optic tubercle and the OL (Figure 15). This contralateral innervation pattern makes it different from the C4 cluster of the bee, although both provide dopaminergic signaling to visual areas of the brain.

The third cell type, but not the first or second, was labeled by both the mono-and polyclonal antibodies we used, and yielded the same labeling pattern. This labeling pattern is notable and is indicative of a dopaminergic modulation of visual circuits and information processing from the lamina to the lobula. It is worth noting that visual forms of aversive learning have been shown in the honey bee, which may depend on dopaminergic signaling in associating the visual chromatic/achromatic stimuli with the aversive electric shock (Mota et al., 2011a). Although the critical coincidence of the visual stimulus and the shock pathways necessary to support visual aversive learning and memory may occur at the level of the MBs and/or CX, such integration could occur at multiple levels upstream of these structures, thus providing multiple substrates for different forms of visual plasticity.

### TH-immunoreactivity in the Mushroom Body

The MB is a higher order processing center, which integrates various types of sensory information conveyed by visual, olfactory, mechanosensory and gustatory inputs (Strausfeld, 2002). Dopaminergic processes innervate the calyces, the vertical and MLs and the PED of the MB. TH-ir in the calyces is observable in the lip and the collar regions, known input regions of olfactory and visual afferents, respectively. The vertical and MLs, sites associated with memory retrieval (Cano-Lozano et al., 2001), are variably innervated by dopaminergic processes.

In the fruit fly *Drosophila melanogaster*, studies on olfactory-based aversive and appetitive learning have revealed that several classes of dopaminergic neurons provide distinct forms of reinforcement signals (appetitive, aversive, short-term, long-term), thus resulting in multiple forms of memories (Claridge-Chang et al., 2009; Aso et al., 2010, 2012; Burke et al., 2012; Liu C. et al., 2012). For example, the PAM cluster that has resemblance to the C1 and C2 clusters, mentioned above, mediates the aversive reinforcement properties of the electric shock used as a US in olfactory aversive conditioning (Claridge-Chang et al., 2009; Aso et al., 2010, 2012).

Despite its relatively small number of neurons (Mao and Davis, 2009), the *Drosophila* PPL1 cluster, which resembles the C3 cluster of the honey bee, provides aversive reinforcement signaling and regulates levels of anesthesia-resistant memory (ARM), and gating to stabilized long-term memory (LTM; Claridge-Chang et al., 2009; Aso et al., 2010; Placais et al., 2012). Moreover, neurons in the PAM and PPL1 clusters may interact at the level of the MBS and tune the stability of aversive memory (Aso et al., 2012). Collectively, at least three DA pathways to the MB can induce aversive (i.e., shock-induced) memory in the fruit fly. The projections arborize in different MB subdomains defined by specific combinations of intrinsic and extrinsic neurons (Aso et al., 2012).

Dopaminergic signaling also mediates appetitive-reinforcement in the fruit fly. It has been recently shown that sucrose reinforcement is mediated by a hierarchical network in which peripheral signaling is mediated by octopaminergic neurons that further convey their signal to dopaminergic neurons within the PAM cluster and on to the MBs (Burke et al., 2012; Liu C. et al., 2012). Thus, a different pathway of dopaminergic signaling indicates the presence of reward in the formation of appetitive memory (Burke et al., 2012; Liu C. et al., 2012).

Results from the fruit fly have underscored the fundamental importance of different subsets of dopaminergic neurons from the PAM and PPL1 clusters, and thus serving as neural correlates of reinforcement signaling in appetitive and aversive olfactory conditioning. In the bee, appetitive reinforcement appears to be independent of dopaminergic signaling as it is mediated by a single octopaminergic neuron, the VUMmx1 neuron, which arborizes in the ALs, LH and MBs, and whose activity substitutes for sucrose reward in appetitive olfactory conditioning (Hammer, 1993). Yet, the dependency of aversive olfactory SER conditioning on dopaminergic signaling has been demonstrated using pharmacological blockade (see “Introduction” Section; Vergoz et al., 2007). The specific neurons mediating the shock signaling in this aversive conditioning paradigm might just be found in the C1, C2 and/or C3 clusters given the apparent homologies with the PAM and PPL1 clusters of the fruit fly.

### TH-immunoreactivity in the Central Complex

The CX of the bee is a structure made of four interconnected, midline spanning neuropils: the upper and LDs of the CB, the PB located more posteriorly, and a pair of ventral NO (Kenyon, 1896; Jonescu, 1909). The CX is involved in different functions such as sensory integration, motor control, spatial learning and sensorimotor integration (Pfeiffer and Homberg, 2014). It is particularly important for the processing of visual information (Homberg, 1985; Milde, 1988). We show that dopaminergic innervation of the CX can be attributed to at least two clusters, the C3 cluster that innervates the anterior UD of the CB, and the C3b cluster, which projects to the posterior upper and LDs of the CB and NO. The somata of the C3b cluster were located in the posterior region anterior to the PB. Interestingly, the PPM3 cluster in *Drosophila* is also located in a relatively similar region with a similar number of somata (eight; see Mao and Davis, 2009). In the fly, these projections can be traced to the lower half of the fan-shaped body, the NO (Mao and Davis, 2009; Alekseyenko et al., 2013) and the ellipsoid body (Liu Q. et al., 2012).

The presence of dopaminergic neurons in the CX of *Drosophila* has been associated with sleep, arousal, wakefulness and aggression (Ueno et al., 2012; Alekseyenko et al., 2013). So far, dopaminergic processes in this neuropil have not been associated with reinforcement-signaling functions for appetitive and/or aversive associative learning and memory. This finding may be due to the fact that conditioning protocols in which dopaminergic function has been studied in the fly are mostly olfactory. In contrast, conditioning protocols that involve visual patterns associated with the aversive reinforcement of heat on the thorax (Wolf et al., 1992) do involve groups of horizontal neurons in a substructure of the CX that is required for *Drosophila* visual pattern memory (Liu et al., 2006). In addition, a small set of neurons in the ellipsoid body, another substructure of the CX and connected to the fan-shaped body, is also required for visual pattern memory. Both groups of neurons thus constitute a complex neural circuit in the CX for *Drosophila* visual pattern memory (Pan et al., 2009), which may benefit from a possible association with dopaminergic circuits conveying aversive reinforcement signaling.

### TH-immunoreactivity in the Antennal Lobe

The AL and AMMC are prominent neuropils in the bee brain. The AL is the primary olfactory neuropil and, in the honey bee, it comprises *ca*. 160 globular subunits termed Glo. Glo are interaction sites primarily between the afferent projections of olfactory receptors on the antenna, local interneurons connecting glomeruli laterally, and projection neurons conveying olfactory inputs to higher-order centers such as the LH and the MB; efferent modulatory projections are also associated with Glo. The AMMC receive mainly mechanosensory input from the antennae and house antennal motoneurons (Pareto, 1972; Suzuki, 1975).

Our anti-TH labeling revealed dopaminergic projections in the AL, which could be traced back to two small clusters, S1 and S2, located in the SEZ. Because dopaminergic signaling is vital for aversive olfactory conditioning in bees (Vergoz et al., 2007), the presence of TH immunoreactive fibers in the AL may indicate that dopaminergic modulation is important for learning or olfactory plasticity upstream of the MBs and the LH.

### TH-immunoreactivity in the Subesophageal Zone

The SEZ is a fused region containing the mandibular, maxillary and labial neuromeres. In the honey bee, as in other insects, the suboesophageal ganglion gives rise to motoneurons of the mouthpart muscles and receives sensory (e.g., gustatory) neurons from the mouthparts, mediating the proboscis extension reflex (Rehder, 1988). It processes the gustatory and mechanosensory input from the proboscis and thus, seems to be particularly important for gustatory coding (Rehder, 1988; Marella et al., 2006; de Brito Sanchez et al., 2007). These projections form various tracts: longitudinal, transverse (commissures) and midline; sensory nerve roots are also observed. In the bee, this region is important for associative appetitive learning as it contains the cell body of an important modulatory neuron involved in olfactory appetitive conditioning, the VUMmx1, which substitutes for sucrose in appetitive olfactory conditioning (Hammer, 1993).

Including the S1 and S2 clusters innervating the AL, we found 18 somata in the SEZ, two of which correspond to the ventral unpaired medial (VUM) neurons. These two dopaminergic VUM neurons belong to the novel cluster, S8, revealed by our work. In two other insect models, *Drosophila* and *Calliphora*, six dopaminergic somata have been identified in the SEZ (Nässel and Elekes, 1992; Friggi-Grelin et al., 2003), two of which are VUM neurons. It is therefore possible that the S8 cluster correspond to these neurons existing in flies.

Unfortunately, we were unable to distinguish clearly the processes coming from the S7 and the S8 clusters. Their arborizations appear to innervate dorsal regions bordering the SPZ where the motoneurons that control the movement of the mouthparts are located (Rehder, 1989). The dopaminergic VUM neurons found in our work might be of particular interest in the context of appetitive learning. Recently, a dopaminergic VUM neuron with extensive branching in the SEZ has been shown to trigger proboscis extension in *Drosophila*, and to have an activity that is altered by satiety state (Marella et al., 2012).

### Dopaminergic Neurons as Modulators of Behavior

Besides their role in reinforcement signaling, dopaminergic neurons act as a more global modulatory system, generally depressing several behavioral components. For instance, DA decreases sucrose responsiveness (i.e., PER to increasing sucrose concentrations) when injected into the thorax. Also, injection or feeding of the DA receptor agonist 2-amino-6,7-dihydroxy-1,2,3,4-tetrahydronaphthalene (6,7-ADTN) reduces sucrose responsiveness significantly (Scheiner et al., 2002). In olfactory PER conditioning, injection of DA into the ALs significantly reduces olfactory retention after one and three conditioning trials (Macmillan and Mercer, 1987). In the case of aversive responsiveness (i.e., SER to increasing shock voltages), dopaminergic blockade induces an increase of shock responsiveness, thus reflecting an enhancement of shock sensitivity (Tedjakumala et al., 2014). This result thus indicates that in its default mode, and besides its reinforcement-signaling role, dopaminergic signaling acts as a depressor of sting responsiveness to electric shocks so that when its effect is antagonized, responsiveness increases (Tedjakumala and Giurfa, 2013).

A possible explanation for this dual function is to assume the existence of different classes of dopaminergic neurons mediating different functions: one class acting as a general gain control system, with the specific role of down-regulating responsiveness and another class acting as instructive neurons in aversive associative learning, mediating aversive US signaling. Owing to these different functions, their brain targets could be different. While the first class would exhibit extensive and broad branching within the entire brain in order to modulate different motivational components (appetitive, aversive) and sensory modalities (olfactory, visual gustatory, etc.), the second class would exhibit a specific connectivity with respect to CS-processing circuits (e.g., olfactory, visual) in order to facilitate CS-US associations and provide instructive (i.e., valence) information to the targeted CS circuit (Giurfa, 2006). Although further studies are clearly warranted to address the possible heterogeneity of different dopaminergic clusters in the honey bee brain, in principle, the neural architecture of the dopaminergic circuits we have described in the present work provides a solid foundation for future discovery and identification of these various functions.

## Ethics Statement

Experiments on honey bees are not subject to the approval of ethics committee. All experiments were nevertheless performed taking care of ethic procedures and minimizing the number of animals required for data gathering.

## Author Contributions

All authors had full access to all the data in the study and take responsibility for the integrity of the data and the accuracy of the data analysis. SRT and MG: study concept and design; drafting of the manuscript. SRT, JR, M-LB and KAM: acquisition of data. LH and IM: support for data acquisition. SRT, JR, M-LB and MG: analysis and interpretation of data.SRT, JR, KAM and MG: critical revision of the manuscript for important intellectual content.MG: obtained funding; study supervision. SRT, M-LB, JR, IM and LH: administrative, technical and material support.

## Conflict of Interest Statement

The authors declare that the research was conducted in the absence of any commercial or financial relationships that could be construed as a potential conflict of interest.

## Abbreviations

adpc: anterior dorsal protocerebral commisure
aiot: anterior inferior optic tract
AL: antennal lobe
AMMC: antennal mechanosensory and motor center
AOTU: anterior optic tubercle
asot: anterior superior optic tract
CA: calyx
CB: central body
CX: central complex
DA: dopamine
ES: esophagus
Glo: glomeruli
ir: immunoreactivity
LbDC IV: labial dorsal commissure IV
LbSMC: labial superior median commissure
LbVC II: labial ventral commissure II
l-CA: lateral calyx
LH: lateral horn
MB: mushroom body
MBEN: mushroom-body extrinsic neuron
m-CA: median calyx
ML: medial lobe
MVT: medial ventral tract
MxMT: maxillary midline tract
NO: ventral noduli
OL: optic lobe
PAL cluster: protocerebral anterior lateral cluster
PAM cluster: protocerebral anterior medial cluster
PB: protocerebral bridge
PED: pedunculus
PEDN: pedunculus neck
PPL cluster: protocerebral posterior lateral cluster
PPM cluster: protocerebral posterior medial cluster
SER: sting extension reflex
SEZ: subesophageal zone of the brain
SPZ: supraesophageal zone of the brain
TH: tyrosine hydroxylase
VIT: ventral intermediate tract
VL: vertical lobe
VLT: ventral lateral tract
VMT: ventral medial tract.
